# Nonfatal injury emergency department visits and inpatient hospitalizations among persons under age 65 with an intellectual and developmental disability or deaf or hard of hearing disability

**DOI:** 10.1186/s40621-025-00580-2

**Published:** 2025-05-09

**Authors:** Terry L. Bunn, Jacqueline Seals, Dana Quesinberry, Alaina Murphy, Julia F. Costich

**Affiliations:** 1 Department of Epidemiology and Environmental Health, University of Kentucky College of Public Health, Lexington, KY USA; 2 Kentucky Injury Prevention and Research Center, University of Kentucky College of Public Health, Lexington, KY USA; 3 Department of Health Management and Policy, University of Kentucky College of Public Health, Lexington, KY USA

## Abstract

**Background:**

Vulnerable populations at risk of injury include persons with intellectual and developmental disabilities (IDD), and persons who are deaf or hard of hearing (DHH). The purpose of this study was to describe and compare emergency department and inpatient hospitalization (ED + IP) injury rates and rate ratios by injury type among persons under age 65 with IDD or with DHH to those without IDD or DHH.

**Methods:**

This is a descriptive population-based retrospective cross-sectional study of injuries among patients under the age of 65 with an IDD disability or a DHH disability using Kentucky ED + IP discharge datasets from 2019 to 2023. Injury rates and injury rate ratios were calculated for those under the age of 65 with an IDD or a DHH disability and without an IDD or a DHH disability, using number of persons under age 65 with or without the related disability as the denominator.

**Results:**

The overall injury rate was lower for persons under age 65 with an IDD or DHH compared to those without those disabilities in 2023 (1 and 3 per 100,000 population, respectively). IDD or DHH disability types had significantly lower overall ED + IP injury rate ratios compared to those without those disabilities (IDD: 0.667 [95% CI: 0.640–0.694], DHH: 0.658 [95% CI: 0.633–0.683]). When ED + IP injury type rate ratios were compared, IDD or DHH persons had higher injury rate ratios for self-harm (IDD: 8.740 [95% CI: 7.783–9.815], DHH: 1.7846 [95% CI: 1.402–2.272]), assault (IDD: 1.386 [95% CI: 1.173–1.637], DHH: 1.310 [95% CI: 1.115–1.540]), unintentional falls (IDD: 1.540 [95% CI: 1.436–1.633], DHH: 1.283 [95% CI: 1.201–1.372]), and drug poisonings (IDD: 2.401 [95% CI: 2.103–2.740], DHH:1.620 [95% CI: 1.391–1.886]) compared to those without such disabilities. Those with IHH or DHH who were treated for injuries incurred triple the charges of patients without these conditions (~$17,086 IDD; $19,550 DHH; and $5,216 no IDD or DHH disabilities).

**Conclusions:**

These findings have implications for health policy at the state and federal level. Clinical care interventions to reduce assault, self-harm, drug poisonings and unintentional injuries and healthcare utilization in persons with IDD or DHH should be publicly funded or covered by health insurance.

## Introduction

Injuries take an enormous toll on the US population in terms of financial, physical, and mental well-being. According to the National Safety Council, 63 million persons were injured and treated in 2022, a 162% increase over the last 30 years [22]. In 2021, the associated costs of injuries totaled approximately $1.3 billion [22].

Injuries affect vulnerable populations such as persons with intellectual and developmental disabilities (IDD), and those who are deaf or hard of hearing (DHH). In the United States, it has been estimated that 28.7% of all adults have a disability; 13.9% have a cognition disability and 6.2% have a hearing disability [8]. The increased risk of physical injury among children with an IDD is well-documented, but data on non-elderly adults with IDD has not been as well-studied. IDD is associated with increased prevalence of many chronic conditions [31], so it is not surprising that injuries are also more common in this population. Calver et al. [6] found a 1.79 higher rate of hospitalized intentional and unintentional injuries in IDD compared with non-IDD children and adolescents. However, a prospective cohort study found the increase in injury prevalence among young people with IDD was limited to those who also had attention deficit-hyperactivity disorder [29]. A particularly concerning finding regarding non-accidental trauma (NAT) by Baghdadi et al. [4] was that children with IDD were 9 times as likely to be hospitalized with NAT injuries than those without IDD. Similarly, Samuel and colleagues [24] found that children with developmental disabilities were over-represented among children who died because of abusive injuries.

This phenomenon is not limited to children and adolescents. For example, a Swedish study documented increased risk of injury in adults aged 65 and older with autism and of fall-related injury in older adults with IDD [17]. In a study of U.S. emergency department (ED) visits by IDD status and race or ethnicity, the authors found that persons with IDD had higher rates of multiple chronic condition-related ED visits [31]. They also showed that Black and Latinx persons with IDD had higher rates than whites for multiple chronic condition ED visits.

Studies utilizing ICD-9-CM codes or registration with a general practitioner/family physician to identify persons with IDD, and using varied data sources have been conducted. Emergency department (ED) visits for injuries among persons with IDD was 1.5 times higher than the general population in a study using Medicaid Claims data from eight states [30]. In a study of women with IDD in Massachusetts using longitudinal individual-level data from multiple linked data sources, [20] found that the prevalence of ED visits (with and without injuries) for post-partum women was three times higher among women with IDD compared to women without IDD. Last, a study by Finlayson et al. [10] in Scotland utilizing self-reported surveys found that adults with IDD had an elevated rate of injuries including falls when compared to the general population.

Unintentional injury is less studied in the DHH population, but several investigations have identified heightened risk of self-harm in this population [28]. Focus on the non-elderly DHH population is important because the high prevalence of hearing impairments in older adults is a confounding factor in analyses of their relationship to injury rates and types. It is also important to note that third-party coverage for assistive hearing devices is widely available for children and adolescents but rarely so for older adults, so individuals coded as DHH in our data may not live with serious functional impairments.

The purpose of this study was to describe and compare ED + inpatient hospitalization (IP) injury rates among persons under age 65 with IDD or DHH by injury type and other variables with ED + IP rates among those without either disability using ICD-10-CM coded data to identify persons with IDD and DHH. We hypothesized that rates of ED visits and IP for injuries of persons with IDD or DHH would be higher compared with rates of ED visits and IP for injuries of persons without either disability. The IDD and DHH groups were chosen for study because their representation in Kentucky ED and IP records was robust enough to support detailed analysis.

## Methods

We conducted a descriptive population-based retrospective cross-sectional study using combined ED and IP patient encounter data.

### Data sources

Data for this study were extracted from IP and ED discharge datasets obtained from the Office of Data Analytics in the Secretary’s Office of the Cabinet for Health and Family Services. These datasets include information on all IP and ED patients treated at Kentucky hospitals, with data available through 2023. The Kentucky Injury Prevention and Research Center, in its role as a bona fide agent for the Kentucky Department for Public Health, provided these datasets to the investigators. To avoid duplication of cases in the combined ED and IP dataset, encounters that were discharged from an IP admission were removed from the ED dataset. Fatal encounters were also removed from the dataset and were not analyzed for this study.

### Study population

The study population included all nonfatal injury-related ED + IP encounters in Kentucky hospitals involving Kentucky residents 64 years and younger. Patient encounters were classified by disability status:

1. Intellectual and Developmental Disabilities (IDD) or.
2. Deaf and Hard of Hearing (DHH) or.
3. no IDD or DHH recorded.

Nonfatal injury-related encounters were defined using ICD-10-CM codes, following guidance from the Council of State and Territorial Epidemiologist Surveillance (CSTE) Toolkit [13, 14]. IDD was defined using the following ICD-10-CM codes: F70-F73, F78-F79, F81, F849, and Z736. DHH was defined using ICD-10-CM codes H90-H91. The group without the targeted disabilities was identified by excluding all cases with the ICD-10-CM IDD or DHH disability code groups.

### Variable selection

Demographic variables that were analyzed included age group, biological sex, race, ethnicity, metro status, Appalachian status, injury type, expected payer, and location of treatment. Averages of total charges by disability status group were calculated. Age groups included 0–17, 18–34, and 35–64 years. Race was identified as black, white or other. Ethnicity was defined as either Hispanic or non-Hispanic, and biological sex was identified as either male or female. Metro status was based on the Rural-Urban Continuum Codes (RUCC) defined as a residence in either a metropolitan (RUCC 1–3) or non-metropolitan area (RUCC 4–9). Appalachian Status was defined as resident counties served by the Appalachian Regional Commission [1]. Expected payer was categorized into Medicare, Medicaid, Commercial, Self-Pay/Charity, and ‘other’ categories. Location of treatment was identified from the discharge status as either ED or IP.

Self-harm, assault, unintentional fall, unintentional motor vehicle crash, and drug poisoning (regardless of intent) injuries were identified using ICD-10-CM codes, following guidance from the CSTE Surveillance Toolkit [13, 14]. The CSTE Surveillance Toolkit does not include definitions for struck by/against (regardless of intent) and overexertion injuries. Therefore, struck by/against and overexertion injuries were defined using the National Center for Health Statistics External Cause of Injury Matrix [12].

### Statistical analysis

Population-based injury rates for the year 2023 were calculated as the number of injuries per 1000 disability population group and by age. The denominator numbers for persons with or without the related disability were derived from the 1-year estimates from the American Community Survey disability characteristics table [7].

ED + IP injury rates for the years 2019–2023 were calculated as injury encounters per total number of ED + IP encounters for each disability status. Rates were stratified by the disability and demographic variables. Rates for the stratified disability were calculated for diagnosed injury types, including visits for: self-harm; assaults; unintentional injuries (falls, motor vehicle crashes); injury mechanism regardless of intent (overdoses and struck by/against); and overexertion as well as other hospital or care specific information. Rates were calculated using the following equation:

$$\begin{aligned}&( {Injury\:Encounters{\text{ }}( {per\:disability\:status} )})/\\&\qquad( {All\:hospital\:encounters}\: \\&\qquad{( {per\:disability\:status})} )*1,000\end{aligned}$$

Demographic specific rate ratios were calculated to compare injury outcomes among demographic groups and injury types in each disability group, with the group with neither IDD nor DHH being the reference group. Demographic specific rate ratios and 95% confidence intervals were calculated by demographic/injury categories. P-values were also calculated to determine significance. Demographic specific rate ratios were calculated comparing rates of injury between disability status groups and visits where an IDD or DHH disability were not identified.

Demographic specific rate ratios were calculated for each group using the following equation:

$$\begin{aligned}&( {Demographic\:specific\:injury\:rate\:for\:males\:in\:}\\&\qquad{IDD\:group} )/( {Demographic\:specific\:injury\:}\\&\qquad{rate\:males\:in\:no\:disability\:group\:}\\&\qquad{( {reference\:group} )} ) \end{aligned}$$

Confidence Intervals for rate ratios were calculated using the following equation:

$$95\% {\text{ }}CI\,=\,Rate{\text{ }}ratio{\text{ }} \times {\text{ }}exp\left( { \pm \,1.96 \times SE\left( {log} \right)RR} \right)$$

$$Log\left( {RR} \right)\,=\,natural{\text{ }}log{\text{ }}of{\text{ }}rate{\text{ }}ratio$$

$$SE\,=\,Standard{\text{ }}error{\text{ }}of{\text{ }}the{\text{ }}log{\text{ }}rate{\text{ }}ratio$$

All analyses were conducted using SAS version 8.16 for Windows (SAS Institute Inc).

## Results

The 2023 rate of injuries receiving hospital-based care was lower for persons with IDD or DHH (1 and 3 per 100,000 population, respectively) compared to those without such disabilities (106 per 100,000 population) (Table 1). Injury rates were highest in the 0–17 and 18- 34-year age categories for the DHH group (10 and 6 per 100,000 population, respectively) compared to those age categories for the IDD group (1 and 2 injuries per 100,000 population).

**Table 1:** Nonfatal emergency department (ED) visit and inpatient hospitalization (IP) injury rates by disability status in Kentucky, 2023

	Injuries Treated in ED and Inpatient Hospitalizations		
	Intellectual and Developmental Disabilities	Deaf or Hard of Hearing Disabilities	No Intellectual and Developmental, Deaf or Hard of Hearing Disabilities
**Number of Injuries**	443	314	351,885
	**Rate (Number of Injuries/1000 disability population group)**		
**Overall Injury Rate**	1	3	106
**Age Group**			
0–17	1	10	110
18–34	2	6	112
35–64	2	2	101

The highest ED + IP injury rate was for the 0- 17-year age category in persons without IDD or DHH (253 per 1000 neither disability group) (Table 2). The lowest injury rate was for the 35- 64-year age category in DHH persons (111 per 1000 DHH group). Comparing the two disability groups, the DHH group had elevated injury rates for the younger (196 per 1000 DHH group vs. 123 per 1000 IDD group) and middle age groups (173 1000 DHH group vs. 140 per 1000 IDD group). The IDD group, in contrast, had a higher injury rate in the 35–64-year age category than the DHH group of the same age (130 per 1000 IDD group vs. 111 per 1,000 DHH group). Males overall had higher injury rates than females in all three groups (134 vs. 131 per 1000 IDD group, 138 vs. 124 per 1000 DHH group, 236 vs. 170 per 1000 neither disability group).

Regarding race, the highest ED + IP injury rate was for Whites without IDD or DHH (204 per 1000 disability group) (Table 2). Blacks and ‘other’ races had elevated injury rates in the DHH group (127 and 151 per 1000 DHH group) compared to Blacks and ‘other’ races in the IDD group (98 and 99 per 1000 IDD group). Overall, injury rates were higher for non-Hispanics but DHH Hispanics had a higher injury rate than Hispanics with IDD (117 per 1000 DHH group vs. 108 per 1000 IDD group).

Metro and non-metro ED + IP injury rates were lower for those with both types of disability compared to those without them (Table 2). Non-Appalachian and Appalachian injury rates were elevated for those without IDD or DHH (200 and 198 per 1000 neither disability group, respectively) although the Appalachian IDD group had a higher injury rate compared to the Appalachian DHH group (135 per 1000 IDD group vs. 117 per 1000 DHH group).

**Table 2:** Demographics of nonfatal injury emergency department (ED) visits and inpatient hospitalizations (IP) by disability status in Kentucky, 2019–2023

	Injuries Treated in ED and IP		
	Intellectual and Developmental Disabilities	Deaf or Hard of Hearing Disabilities	No Intellectual or Developmental, Deaf or Hard of Hearing Disabilities
**Number of Injuries**	2414	2700	1,735,237
	**Rate (Number of Injuries/1**,**000 Disability Group ED + IP)**		
**Overall Injury Rate**	133	131	199
**Age Group (in Years)**			
0–17	123	196	253
18–34	140	173	193
35–64	130	111	178
**Sex**			
Female	131	124	170
Male	134	138	236
**Race**			
Black	98	127	179
White	138	131	204
Other	99	151	175
**Ethnicity**			
Hispanic	108	117	169
Non-Hispanic	133	131	201
**Metro Status**			
Metro	139	137	196
Non-Metro	126	122	204
**Appalachian Status**			
Non-Appalachian	131	136	200
Appalachian	135	117	198

The highest injury causal rate for all three groups was for unintentional falls (IDD: 385, DHH:323, neither disability group: 251 per 1000 disability group) (Table 3). The self-harm injury rate was highest for the IDD group (120 per 1000 IDD group) compared to the other two groups (24 per 1000 DHH group, 14 per 1000 neither disability group). The unintentional motor vehicle crash rate was elevated among the DHH group (127 per 1000 DHH group) compared to the other two groups (36 per 1000 IDD group, 119 per 1000 neither disability group). Last, drug poisoning was highest for persons with IDD (91 per 1,000 IDD group) and lowest for those without the targeted disabilities (38 per 1000 neither disability group).

The average charges for injuries in the IDD and DHH groups ($17,086 and $19,550, respectively) were more than triple the average charges for those without ($5,216) (Table 3). Medicare was the most frequent expected payer for injuries in the IDD group (excluding ‘other’) (142 per 1000 IDD group), whereas self-pay or charity was the most frequent expected payer for the DHH group (153 per 1000 DHH group) and for those without the related disabilities (226 per 1000 neither disability group). Not surprisingly, the injury rates were higher in the ED for all three groups (IDD: 223, DHH: 201, neither disability group: 230 per 1000 disability group) compared to the IP (IDD: 56, DHH:52, neither disability group: 36 per 1000 disability group).

**Table 3:** Injury characteristics of nonfatal injury emergency department (ED) visits and inpatient hospitalizations (IP) by disability status in Kentucky, 2019–2023

	Injuries Treated in ED and IP		
	Intellectual and Developmental Disabilities	Deaf or Hard of Hearing Disabilities	No Intellectual and Developmental, Deaf or Hard of Hearing Disabilities
	**Rate (Number of Injuries/1**,**000 Disability Group ED + IP)**		
**Injury Type**			
Self-harm	120	24	14
Assault	58	54	42
Unintentional fall	385	323	251
Unintentional motor vehicle crash	36	127	119
Drug poisoning	91	61	38
Struck by/Against	106	119	132
Overexertion	16	45	69
**Expected Payer**			
Medicare	142	105	144
Medicaid	124	134	184
Commercial	118	139	216
Self-Pay/Charity	140	153	226
Other	192	239	421
**Average Charges**	$17,086	$19,550	$5,216
**Location of Treatment**			
ED	223	201	230
IP	56	52	36

Both disability groups had lower overall ED + IP injury rate ratios (IDD: 0.667 [95% CI: 0.640–0.694], DHH: 0.658 [95% CI: 0.633–0.683]) compared to those without the targeted disabilities (Table 4). Both disability groups also had lower injury rate ratios by age group, sex, ethnicity, metro status, and Appalachian status compared to those without those disabilities.

**Table 4:** Rate ratios for nonfatal injuries by disability status and demographics in Kentucky, 2019–2023

			Stratified Model					
	Overall Model		Intellectual and Developmental Disabilities		Deaf or Hard of Hearing Disabilities		No Intellectual and Developmental, Deaf or Hard of Hearing Disabilities (Reference Group)	
	Adjusted Rate Ratio (95% CI)	p-value	Adjusted Rate Ratio (95% CI)	p-value	Adjusted Rate Ratio (95% CI)	p-value	Adjusted Rate Ratio (95% CI)	p-value
**Disability Status**								
Overall	0.999 (0.996–1.001)	0.169	0.667 (0.640–0.694)	< 0.001	0.658 (0.633–0.683)	< 0.001	1.000 (0.998–1.002)	1.000
**Age Group**								
0–17	0.999 (0.995–1.003)	0.726	0.484 (0.420–0.556)	< 0.001	0.774 (0.710–0.845)	< 0.001	1.000 (0.996–1.004)	1.000
18–34	0.999 (0.995–1.003)	0.719	0.726 (0.678–0.778)	< 0.001	0.895 (0.823–0.974)	0.001	1.000 (0.996–1.004)	1.000
35–64	0.998 (0.995–1.001)	0.222	0.731 (0.694–0.771)	< 0.001	0.622 (0.593–0.653)	< 0.001	1.000 (0.997–1.003)	1.000
**Sex**								
Female	0.999 (0.996–1.002)	0.561	0.774 (0.729–0.822)	< 0.001	0.731 (0.692–0.773)	< 0.001	1.000 (0.997–1.003)	1.000
Male	0.998 (0.995–1.001)	0.131	0.566 (0.995–1.001)	< 0.001	0.583 (0.553–0.614)	< 0.001	1.000 (0.997–1.003)	1.000
**Race**								
Black	0.999 (0.993–1.005)	0.672	0.548 (0.479–0.626)	< 0.001	0.709 (0.629–0.800)	< 0.001	1.000 (0.994–1.006)	1.000
White	0.998 (0.996–1.001)	0.180	0.678 (0.650–0.707)	< 0.001	0.644 (0.618–0.670)	< 0.001	1.000 (0.998–1.002)	1.000
Other	0.999 (0.987–1.012)	0.930	0.562 (0.367–0.862)	0.008	0.859 (0.676–1.091)	0.212	1.000 (0.987–1.013)	1.000
**Ethnicity**								
Hispanic	0.999 (0.988–1.011)	0.915	0.642 (0.422–0.974)	0.037	0.695 (0.537–0.899)	0.006	1.000 (0.989–1.011)	1.000
Non-Hispanic	0.999 (0.996–1.001)	0.164	0.664 (0.638–0.691)	< 0.001	0.655 (0.631–0.681)	< 0.001	1.000 (0.998–1.002)	1.000
**Metro Status**								
Metro	0.999 (0.996–1.002)	0.347	0.708 (0.672–0.747)	< 0.001	0.699 (0.667–0.733)	< 0.001	1.000 (0.997–1.003)	1.000
Non-Metro	0.998 (0.995–1.002)	0.318	0.617 (0.580–0.656)	< 0.001	0.598 (0.562–0.637)	< 0.001	1.000 (0.997–1.003)	1.000
**Appalachian Status**								
Non-Appalachian	0.999 (0.996–1.001)	0.266	0.659 (0.626–0.693)	< 0.001	0.680 (0.652–0.710)	< 0.001	1.000 (0.998–1.003)	1.000
Appalachian	0.998 (0.995–1.002)	0.418	0.681 (0.638–0.727)	< 0.001	0.588 (0.542–0.637)	< 0.001	1.000 (0.996–1.004)	1.000

When ED + IP injury type rate ratios were compared, persons with IDD and persons with DHH had higher injury rate ratios for self-harm (IDD: 8.740 [95% CI: 7.783–9.815], DHH: 1.7846 [95% CI: 1.402–2.272]), assault (IDD: 1.386 [95% CI: 1.173–1.637], DHH: 1.310 [95% CI: 1.115–1.540]), unintentional falls (IDD: 1.540 [95% CI: 1.436–1.633], DHH: 1.283 [95% CI: 1.201–1.372]), and drug poisonings (IDD: 2.401 [95% CI: 2.103–2.740], DHH:1.620 [95% CI: 1.391–1.886]) compared to those without such disabilities (Table 5). DHH persons had the same unintentional motor vehicle crash rate ratio as those without the disabilities (~ 1.000), whereas those with IDD had a lower unintentional motor vehicle crash rate ratio (0.303 [95% CI: 0.245–0.374]). Lower overexertion and struck by/against injury rate ratios were observed for those with IDD (0.228 [95% CI: 0.166–0.313] and 0.804 [95% CI: 0.711–0.909], respectively), while that group’s struck by/against injury rate ratio was equivalent to that of persons without such disabilities (~ 1.0000).

**Table 5:** Rate ratios for nonfatal injuries by disability status and injury type in Kentucky, 2019–2023

			Stratified Model					
	Overall Model		Intellectual and Developmental Disabilities		Deaf or Hard of Hearing Disabilities		No Intellectual and Developmental, Deaf or Hard of Hearing Disabilities (Reference Group)	
	Adjusted Rate Ratio (95% CI)	p-value	Adjusted Rate Ratio (95% CI)	p-value	Adjusted Rate Ratio (95% CI)	p-value	Adjusted Rate Ratio (95% CI)	p-value
**Injury Type**								
Self-harm	1.012 (0.994–1.030)	0.196	8.740 (7.783–9.815)	< 0.001	1.785 (1.402–2.272)	< 0.001	1.000 (0.982–1.018)	1.000
Assault	1.100 (0.991–1.011)	0.851	1.386 (1.173–1.637)	0.001	1.310 (1.115–1.540)	0.001	1.000 (0.990–1.010)	1.000
Unintentional fall	1.001 (0.997–1.005)	0.587	1.540 (1.436–1.633)	< 0.001	1.283 (1.201–1.372)	< 0.001	1.000 (0.996–1.004)	1.000
Unintentional motor vehicle crash	0.999 (0.993–1.005)	0.782	0.303 (0.245–0.374)	< 0.001	1.064 (0.957–1.183)	0.254	1.000 (0.994–1.006)	1.000
Drug poisoning	1.003 (0.992–1.014)	0.601	2.401 (2.103–2.740)	< 0.001	1.620 (1.391–1.886)	< 0.001	1.000 (0.989–1.011)	1.000
Struck by/Against	0.999 (0.994–1.005)	0.887	0.804 (0.711–0.909)	0.001	0.904 (0.811–1.009)	0.071	1.000 (0.994–1.006)	1.000
Overexertion	0.998 (0.991–1.006)	0.698	0.228 (0.166–0.313)	< 0.001	0.653 (0.547–0.780)	< 0.001	1.000 (0.992–1.008)	1.000
**Location of Treatment**								
ED	0.999 (0.998–1.002)	0.834	0.968 (0.925–1.012)	0.154	0.872 (0.836–0.909)	< 0.001	1.000 (0.998–1.002)	1.000
IP	1.006 (0.994–1.019)	0.309	1.532 (1.408–1.666)	< 0.001	1.423 (1.303–1.554)	< 0.001	1.000 (0.988–1.012)	1.000

## Discussion

This study shows that while IDD and DHH individuals did not experience higher overall rates of injuries, their rate ratios by injury type were significantly elevated for self-harm, assault, unintentional falls, and drug poisonings. This finding is confirmed using nationally representative survey data sources. In a study using National Health Interview survey data, Brophy et al. [5] found that falls were the leading cause of injury regardless of disability status. The odds ratios for suicidal ideation and suicide attempt among individuals with a cognitive disability were found to be higher relative to those with a hearing limitation using National Survey on Drug Use and Health data [19]. Using the same survey data, Park et al. [23]; showed that substance use disorders were prevalent among DHH persons.

The rate ratio for intentional self-harm was higher for those with an IDD compared those with a DHH or those without either disability. Intentional self-harm among individuals with disabilities has been documented to be higher than among individuals without disabilities [5, 9, 19]. In a study that focused on suicide outcomes, Marlow et al. [19] found that those with more complex disabilities and cognitive disabilities were more likely to have an attempted suicide reported vs. suicidal ideation alone. Cree et al. [9] found that individuals with a cognitive disability were more likely to have a diagnosed depressive disorder or to binge drink.

As with self-harm, we found that individuals with IDD were more likely to be treated for a drug poisoning compared to either the DHH patients or those with no disabilities. While our drug poisoning results do not differentiate by intent, other studies have found that individuals with cognitive disabilities or with more than one disability are at higher risk to have an overdose, substance use, substance misuse, or substance disorder [2, 3, 15]. In these same studies, individuals who are DHH also had elevated substance use outcomes [2, 3, 15].

Comparable to other studies on injuries among individuals with disabilities [5, 26, 27], we found rate ratios for falls for individuals with IDD or DHH were elevated. The rate ratio of assault injuries was also higher for those with IDD or DHH compared to those without either disability. Unlike other injury characterizations, the assault rates for both disability types were similar. Studies have shown that regardless of disability type, individuals with disabilities are at greater risk of being victims of violence [11, 16, 18, 28].

Falls, unintentional struck by/against, and motor vehicle crashes were the three highest injury rates in those without either disability and DHH individuals, similar to the leading causes of nonfatal injuries for the general population of all ages, sex, and races treated in the ED in 2022 [21] but unlike those with an IDD. The variation in injury rates between the population without disability and those with an IDD may be attributable to the lower rate of engagement in activities with elevated risks of injury because of their physical, developmental or cognitive limitations [5, 26, 25]. Variations in injury rates from the general population may also be attributable to the perception of risk in the populations with disabilities, the use of aids, or intervention from a caretaker [25].

These findings have implications for health policy at the state and federal level. Supportive policies to reduce injuries in persons with IDD should be considered by publicly-funded programs that are covered by government and commercial health insurance. Policy changes should address requirements to provide screening for self-harm, substance use, trauma, and fall risk during the provision of regular clinical care. Screening policies can lead to identification of risks prior to addressing acute exacerbations in emergency healthcare settings. Polices should also be considered that require tailoring prevention programs to individuals with disabilities. Funding that facilitates increased access to environmental modifications, behavioral health supports, and caregiver assistance seem likely to yield economic benefits in terms of reduction in the costs associated with injuries in IDD and DHH groups. The burdens of disability are well-documented and need not be exacerbated by heightened risk of injury.

### Limitations

There are limitations to this study, predominantly due to the data source. The hospital discharge data is deidentified so we could only identify injury incidence and were not able to control for injured patients who were transferred between hospitals. Also, the data source only contains visits to Kentucky hospitals. We know there is missing data in areas around the border of Kentucky where larger out-of-state hospitals are closer. Since the data set was based on encounters without individual patient identification, we could not calculate rates based on number of persons treated in the ID or IP. Fourth, cases and variables were identified using ICD-10-CM codes, and we know that there could be a small overlap of disability types. Fifth, self-harm ICD-10-CM codes do not indicate intent (intentional or unintentional). Sixth, since this data is encounter-based, individuals not identified to have an IDD or DHH disability may have a different disability unrelated to the visit, potentially skewing the rate ratios. Last, the discharge data is encounter based, and without the ability to follow an individual patient, we may be missing a diagnosed disability if it was not related to the encounter. The use of the ACS disability data may inflate the injury rates but it is thought that the use of discharge data lowered the rate closer to null.

## Conclusions

While individuals with IDD or DHH had statistically significant lower overall rates of injuries compared to those without either disability in 2023, rate ratios by injury type over the five-year study period were elevated among persons with an IDD or DFF. Rate ratios for intentional self-harm, drug poisonings, assault, and unintentional falls were significantly elevated among persons with IDD or DHH compared to those without either disability. Targeted injury prevention education should address populations whose specific disabilities increase their risk of harm. Access to clinical care interventions to prevent self-harm, assault and repeated assault injuries, and unintentional fall programs may reduce injuries, and ED and IP admissions. Education and interventions should be available to the individuals, their caregivers, and their healthcare providers. Environmental modifications, behavioral health supports, and caregiver assistance can reduce costs associated with injuries among persons with IDD or DHH.

## Data Availability

Data is available upon request to the Cabinet for Health and Family Services. Data is deposited in a folder in the University of Kentucky Enterprise Data Warehouse. The data is not open access. The Kentucky Injury Prevention and Research Center is not the owner of the Emergency Department visit or inpatient hospitalization data. All requests for the data should be made to the data owner, the Office of Data Analytics in the Kentucky Cabinet for Health and Family Services.

## Abbreviations

IDD: Intellectual or developmental disability
DHH: Deaf and hard of hearing disabilities
NAT: Non-accidental trauma
ED: Emergency department
IP: Inpatient hospitalization
Log(RR): Natural log of rate ratio
SE: Standard error of the log rate ratio
